# A case of the crossed coronaries

**DOI:** 10.1259/bjrcr.20200143

**Published:** 2020-10-29

**Authors:** Gautam Sen, Alice Veitch, Sergio Nabais

**Affiliations:** 1 Department of Cardiology, Salisbury Hospital, Odstock Rd, Salisbury, United Kingdom; 2 Department of Radiology, Salisbury Hospital, Odstock Rd, Salisbury, United Kingdom

## Abstract

Coronary artery anomalies are rare; however, can be a potential cause of significant morbidity and even mortality. Crossed left anterior descending and left circumflex arteries is a rare finding, and therefore the significance of this finding is not well understood. CT coronary angiography is an excellent non-invasive modality which enables the diagnosis of such abnormalities, and it is likely that with the increased use of CT coronary angiography in cardiology, other similar cases will be diagnosed.

## Case presentation

**Figure 1.**
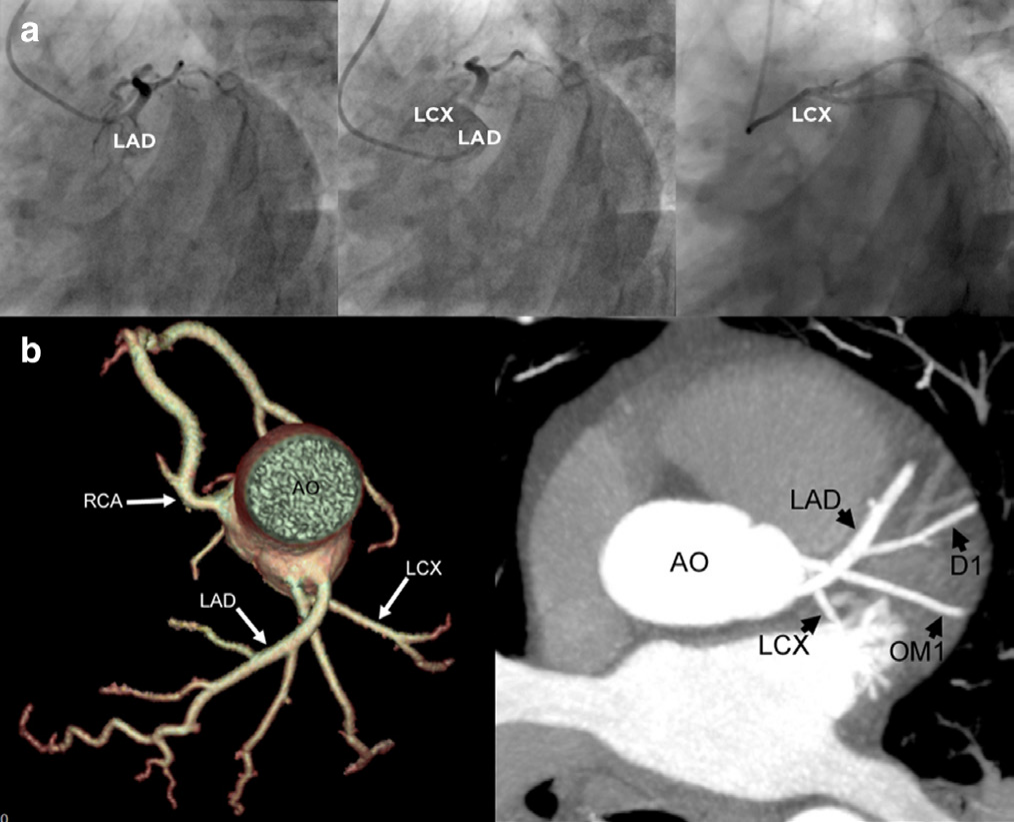
(Panel A) Coronary angiogram images with selective injections of the LAD, non-selective injection to appreciate the anatomical relationship, and selective injection of the LCX. (Panel B) CTCA images with a tree angiogram reformat and axial MIP reformat showing crossing of the LAD and LCX. AO, aorta; D1, diagonal artery; LAD, left anterior descending artery; LCX, left circumflex artery; OM, obtuse marginal artery; RCA, right coronary artery.

A 48-year-old male patient was investigated in cardiology outpatients for a 3 month history of exertional chest pain and breathlessness. He did not have any cardiovascular risk factors and had no family history of coronary artery disease. A 12-lead electrocardiogram showed normal sinus rhythm and transthoracic echocardiography showed normal biventricular systolic function. Coronary angiography was performed which showed separate origins and an unusual initial course of the left anterior descending (LAD) and left circumflex (LCX) arteries (Panel A). The right coronary artery (RCA) was normal. There was however no obstructive epicardial coronary disease. Following the invasive coronary angiogram, computed tomography coronary angiogram (CTCA) was performed to further assess the anomalous left coronary artery anatomy (Figure 1 Panel a).

The patient was imaged on a GE750HD CT scanner, with imaging and contrast protocols adjusted for his body mass index (BMI) and heart rate on breath-hold. Patient preparation included heart rate control with intravenous metoprolol (beta-blocker), and administration of sublingual glyceryl trinitrate (GTN). For the scan, 80 ml of Omnipaque-350 contrast was administered intravenously at 6 ml/s and followed by 50 ml of saline via CT pump injector. The CTCA was acquired prospectively at 75% (diastole) with 80 ms padding and the DLP was 165 mGy-cm.

CTCA showed that the LAD and LCX originated separately from the left coronary cusp and were switched in position, with the LAD running superiorly to the LCX (Figure 1 Panel b). No narrowing was seen at point of vessel crossover. The diagonal (D1) and obtuse marginal (OM) arteries originated as normal from the LAD and LCX, respectively. The patient has been followed up in clinic for a total duration of 2 years. however has not had any intervention to treat the crossed arteries as he has been asymptomatic.

## Discussion

In normal coronary anatomy, the LAD and the LCX do not cross-over, but run parallel to each other, usually with a single left main stem from the left coronary cusp of the aorta (AO). Coronary artery anomalies are uncommon and are observed in approximately 1% of the general population.^1^ These anomalies can be prognostically significant, *e.g.* arteries with an anomalous origin which take an interarterial course while others are not prognostically significant, *e.g.* arteries which take a retroaortic course.

The incidence of cases of crossed coronary arteries has not well been described in the literature and seems to be very low. In fact, crossing of the LAD and LCX arteries has only been reported twice in the literature.^2,3^ The clinical implications of crossing coronary arteries is unclear; however, there are reports suggesting the possibility of vessel compression. As the condition has uncertain prognostic significance, symptomatic patients may need further work up with functional imaging to decide on appropriate treatment.

CTCA is one of the best techniques to give anatomical images of the coronary arteries and therefore for identifying anomalous coronaries arteries.^4^ As CTCA becomes a major modality for cardiac imaging it is likely that more cases of crossed coronary arteries will be reported in the literature, and therefore its significance will be better understood. Even though crossed LAD and LCX is a rare coronary anomaly, cardiologists, cardiac radiologists and cardiac surgeons should be aware of its existence as it may be a prognostically significant condition in certain cases.

## Learning points

1. Anomalous coronary arteries are not common in the general population but can lead to cardiac complications and therefore cardiologists and cardiac surgeons should be aware of their clinical significance
2. Crossing of the coronary arteries is a very rare abnormality which has not been well documented in the literature previously, with only two reports of crossing of the left anterior descending artery and left circumflex artery
3. CTCA is an excellent non-invasive modality which enables the diagnosis of such abnormalities, and over time can help cardiologists and cardiac radiologists appreciate such abnormalities
